# PFKFB4 promotes M2 polarization of tumor-associated macrophages through aerobic glycolysis-mediated modification of histone H3K18 lactylation in hepatocellular carcinoma

**DOI:** 10.1186/s40170-026-00431-8

**Published:** 2026-04-15

**Authors:** Chao Gan, Dan Wu, Yue Yuan, Lingling Li, Jia Bai, Yanmei Xu, Haihong Lv

**Affiliations:** 1 Medicine Laboratory Centre, The First Hospital of Lanzhou University, Lanzhou, Gansu Province 730000 China; 2 Department of Endocrinology, Tangdu Hospital, Fourth Military Medical University, Xi’an, Shaanxi Province 710032 China; 3 Department of Endocrinology, The First Hospital of Lanzhou University, 1# Donggang West Road, Lanzhou, Gansu Province 730000 China

**Keywords:** Hepatocellular carcinoma, Glycolysis, PFKFB4, Tumor-associated macrophages, M2 polarization, H3K18 lactylation, Arginase 1, CD206

## Abstract

**Objective:**

Hepatocellular carcinoma (HCC) poses a substantial health burden globally. We explored the mechanism of PFKFB4 affecting M2 polarization of tumor-associated macrophages (TAMs) in HCC.

**Methods:**

The correlation between PFKFB4 expression and macrophage infiltration was analyzed by TIMER database. HCC and adjacent tissues from 30 HCC patients were collected to analyze the PFKFB4 positive expression rate, and the infiltration percentages of the M1- and M2-phenotype TAMs via immunohistochemistry and flow cytometry. PFKFB4 was knocked down or overexpressed in HCC cells, with cell glycolytic, proliferation, and migration assessed. M0 macrophages were co-cultured with HCC cells, with lactate level and TAM M2 polarization detected. H3K18 lactylation (H3K18la) level in TAMs, and its enrichment on the M2 polarization-related gene promoters (arginase 1 [Arg-1], CD206) were assessed by western blot and ChIP. Arg-1 and CD206 levels were tested by RT-qPCR and western blot. In vivo validations were performed in nude mice.

**Results:**

PFKFB4 expression was increased in HCC, and was correlated with poor prognosis. The positive expression rate of PFKFB4 was positively correlated with M2-polarized TAM infiltration. PFKFB4 was up-regulated in HCC cells, which promoted the proliferation and migration of HCC cells via the glycolytic pathway and stimulated lactate production. Co-culture of TAMs with PFKFB4-overexpressing HCC cells promoted TAM M2 polarization. PFKFB4 stimulated tumor growth by promoting TAM M2 polarization via the glycolysis/lactate/H3K18la pathway in vivo.

**Conclusion:**

PFKFB4 promoted lactate accumulation in the tumor microenvironment via glycolysis, stimulated the H3K18la/M2 polarization regulatory axis in TAMs, thus regulating the immune microenvironment and promoting HCC growth.

**Graphical Abstract:**

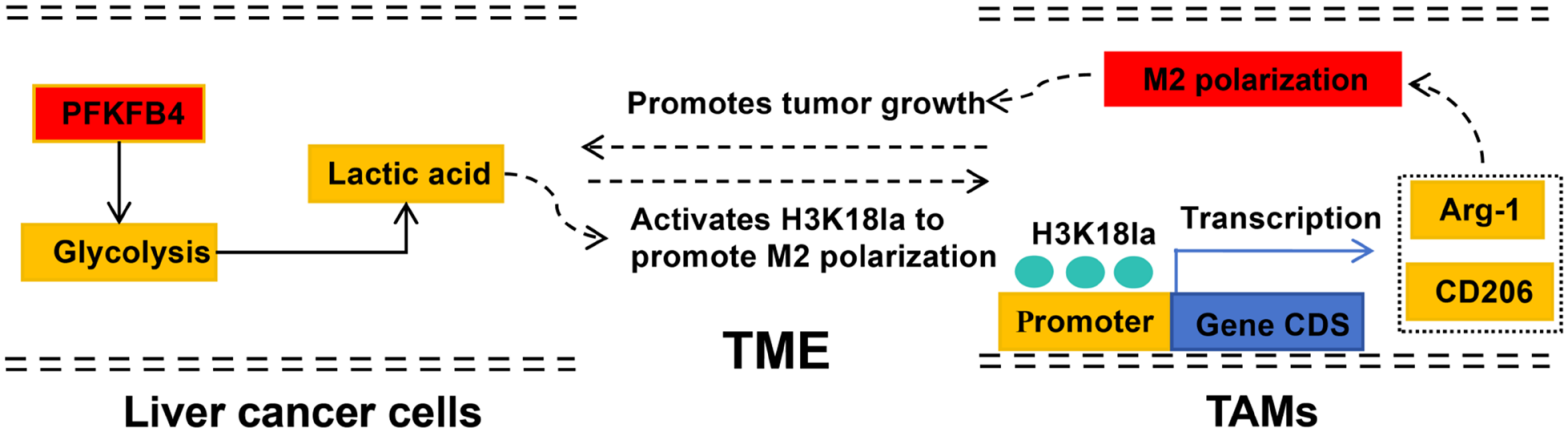

**Supplementary Information:**

The online version contains supplementary material available at 10.1186/s40170-026-00431-8.

## Introduction

Liver cancer is identified as the 6th most common carcinoma throughout the world and the 3rd most leading cause of cancer-associated deaths, with an estimated 906,000 new cases and 830,000 deaths per year [1]. Hepatocellular carcinoma (HCC) accounts for 80–90% of all primary liver cancers [2]. The primary etiologies for HCC include hepatitis C virus and chronic hepatitis B virus infections [3]. Currently, curative treatment regimens for early HCC include radiofrequency/microwave ablation, surgical resection, and liver transplantation [4]. However, HCC recurrence is the key shortcoming of radical treatment, with a 5-year recurrence rate exceeding 70% [5]. Accordingly, it is of great significance to further elucidate the pathogenesis and recurrence mechanism of HCC for improving the therapeutic efficiency and reducing patient mortality.

Tumor microenvironment (TME) represents an ecological niche that supports tumor development and metastasis [6]. Tumor-associated macrophages (TAMs) constitute one of the most abundant infiltrating immune cells in TME, which possess the capacity to advance tumor development, metastasis, and angiogenesis [7, 8]. Importantly, TAMs have emerged as promising treatment targets for diverse cancers, including HCC [9]. Additionally, TAMs are broadly categorized into two distinct functional phenotypes: the pro-inflammatory M1 and the anti-inflammatory M2 subtypes [10]. M1 macrophages have anti-tumor properties with a pro-inflammatory phenotype, whilst M2 macrophages generate tumor-promoting effects, and are featured by an anti-inflammatory phenotype [11]. The status of TAMs is strongly implicated in the prognosis and staging of cancers. In general, early-stage tumors and advanced-stage tumors are associated with anti-tumor (M1) and pro-tumor (M2) properties of TAMs, respectively [12]. Multiple studies have shown that tumor cells can interact bi-directionally with TAMs via TME, thereby regulating TAM polarization to induce them toward a pro-tumorigenic M2 phenotype by secreting signaling molecules encompassing chemokines and cytokines, and that the M2 polarization of TAMs plays a feedback regulatory role in the invasion, proliferation, drug resistance, and immunosuppression in cancer cells [13–15]. However, the mechanism of tumor cells governing the polarization of TAMs through TME has been rarely elucidated.

Glycolytic metabolic reprogramming is perceived as a specific metabolic characteristic of tumor cells, wherein they preferentially generate energy via glycolysis even in the presence of sufficient oxygen, which is known as the Warburg effect or aerobic glycolysis [16]. Aerobic glycolysis is a marker of HCC that participates in the modulation of immune escape, metastasis, invasion, and drug resistance in HCC [17]. Nevertheless, changes in the expression levels of glycolysis-related genes in HCC, as well as the genes mainly affecting glycolytic metabolism in HCC, remain elusive. The research team analyzed the differential expression levels of glycolysis-related genes in HCC through the Cancer Genome Atlas (TCGA) database in the early stage, and identified 6-phosphofructo-2-kinase/fructose-2,6-bisphosphatase (PFKFB) as one of the genes with relatively significantly differential expression. It is noteworthy that PFKFB is a bifunctional enzyme with phosphatase activity and kinase activity that catalyses the synthesis and degradation of fructose-2,6-bisphosphate, and activates the rate-limiting enzyme in glycolysis, phosphofructokinase 1, ultimately facilitating glycolytic fluxes [18]. The PFKFB family is composed of four isozymes, PFKFB1-4 [19]. Among them, PFKFB4 plays a key regulatory role in the glycolytic process, which can modulate glucose metabolism in tumors and modulate glycolytic flux, thereby being involved in the regulation of tumor occurrence and progression [20, 21]. Furthermore, tumor-derived lactate, a by-product of glycolysis, drives TAM polarization toward a pro-tumorigenic M2 phenotype [22]. Nonetheless, whether PFKFB4 affects TAM polarization in HCC by regulating glycolysis remains poorly understood.

Tumor cells cause massive accumulations of lactate in the TME through aerobic glycolysis [22, 23]. The systemic lactate concentration is kept strictly at about 1–2 mM under normal conditions, but the concentration may rise to an abnormal 40 mM within the TME, which affects cellular physiological functions [24]. Another study has suggested that lactate in the TME due to glycolysis may impact the polarization of TAMs. Tumor cell-generated lactate effectively bolsters tumor angiogenesis and growth by facilitating polarization of M2-TAMs and triggering the expression of vascular endothelial growth factor [25]. Tumor-sourced lactate facilitates the polarization of M2 macrophages in breast carcinoma through activating the extracellular signal-regulated kinase/signal transducer and activator of transcription 3 pathway [26]. The evidence indicates that glycolysis-driven lactate accumulation in the TME affects TAM polarization, yet the underlying molecular mechanism remains unclear. Interestingly, lactate induces the modification of histone lysine residues (named lysine lactylation [Kla]). As Kla levels rise, the expression of the M2-like gene *Arg1* increases, promoting the transition of macrophages to an M2 polarization [27]. In addition, methylsulfonylmethane stimulates the Arg1 expression and controls macrophage polarization towards M2 through the lactate/H3K18 lactylation (H3K18la) pathway, thereby alleviating the inflammatory response in a mouse model of peritonitis infection [28]. However, the role of lactate-Kla axis in regulating TAM polarization has yet to be established. As a consequence, this study investigated the role and molecular mechanism of PFKFB4 regulating the polarization of TAMs in HCC via aerobic glycolysis, with a view to providing novel theoretical references for the pathogenesis of HCC, as well as new therapeutic targets and strategies for it.

## Materials and methods

### Ethics statement

This study received endorsement by the academic ethics committee of The First Hospital of Lanzhou University. All tissue specimens were processed with the informed consent of the patient. This study was performed based on the ethical guidelines of the World Medical Association *Declaration of Helsinki*.

### Bioinformatics analysis

Expression and prognosis data for HCC and normal samples were downloaded from the TCGA Genomic Data Commons (GDC) database (https://portal.gdc.cancer.gov/), and the glycolytic pathway dataset was downloaded from the Gene Set Cancer Analysis (GSEA) database (https://www.gsea-msigdb.org/gsea/index.jsp). The expression levels of glycolytic genes in HCC and normal samples were subjected to differential analysis using the R package ‘limma’, with normal samples serving as the control. The *p*-value of the difference was corrected by the false discovery rate (FDR) method. With the criteria of |logFC| > 1 and FDR < 0.05 as the screening criteria for differentially-expressed genes, the glycolytic genes with differential expression were identified. Univariate Cox analysis was implemented on the differentially-expressed glycolytic genes and the prognosis of HCC patients using the ‘survival’ package. Protein interactions of differentially-expressed glycolytic genes were analyzed using the STRING database (https://cn.string-db.org/), and a protein-protein interaction network diagram was established using Cytoscape v3.7.1 software (https://cytoscape.org/), followed by the calculation of the degree value of each gene in the network diagram. The core genes with a degree value > 20 were intersected with the top 20 genes with the highest significance in the results of univariate Cox analysis to eventually obtain the target gene PFKFB4. The differential expression levels of PFKFB4 in normal and HCC samples were analyzed using the GSCA database (http://bioinfo.life.hust.edu.cn/GSCA/#/). Finally, the Kaplan-Meier survival curve was employed for analyzing the relationship between the expression of PFKFB4 and the prognosis of HCC patients. The TIMER database (http://cistrome.shinyapps.io/timer/) was used to analyze the correlation between the expression of PFKFB4 and macrophage infiltration in HCC.

### Tissue collection and prognostic analysis

The primary HCC tissues and adjacent tissue samples were collected from 30 participants who underwent resection in The First Hospital of Lanzhou University from January 2019 to March 2020. The adjacent tissues were defined as the normal liver tissues approximately 3 cm away from the tumor lesions. All patients had received no other treatment prior to surgery, and had complete clinical data. The follow-up data of patients at 3 years postoperatively were obtained, and then the time of death and overall survival of patients were analyzed using Kaplan-Meier survival analysis.

### Immunohistochemistry (IHC)

A portion of tumor tissues and adjacent tissues were fixed overnight at 4 °C in 4% paraformaldehyde (PFA) (P0099-100 mL, Beyotime, Shanghai, China), paraffin embedding, and then cutting into 4 μm-thick sections. The sections were sequentially treated with xylene I, xylene II, anhydrous ethanol I, anhydrous ethanol II, 85% alcohol, and 75% alcohol for deparaffinization and rehydration, immersed in citric acid antigen-retrieval buffer (pH = 6.0) and heated in a microwave oven for antigen retrieval, with endogenous peroxidase activity blocked using H_2_O_2_ treatment. Thereafter, a circle was drawn with a pap pen around the tissues, and the tissues were incubated with 3% bovine serum albumin (BSA; YM-MY563J, Shanghai Yuanmu Bio-Technology Co., Ltd, China) for 1 h to block non-specific binding, followed by incubation with primary antibody anti-PFKFB4 (1/500, ab137785, Abcam, Cambridge, MA, USA) overnight at 4 °C. After phosphate-buffered saline (PBS) washing, the samples were incubated with with immunoglobulin G (IgG) secondary antibody (1/500, ab97051, Abcam) for 2 h at room temperature, followed by color development using diaminobenzidine color developing solution (ab64238, Abcam). After that, the samples were observed and photographed under an optical microscope (Leica, Wetzlar, Germany). The Image J software (National Institutes of Health, Bethesda, MA, USA) was use to quantify the PFKFB4 positive expression rate (%). Five non-overlapping fields of view in each section were randomly observed, with the average value of the results taken.

### Culture of HCC cells

Normal hepatocyte cell line L02 cells (SNL-141) and HCC cell lines PLC/PRF/5 (SNL-086), Huh-7 (SNL-085), Hep3B (SNL-082), and HepG2 (SNL-083) were all acquired from Wuhan Sunn Cell Biotechnology Co., Ltd. (Wuhan, Hubei, China). All cell lines were authenticated by short tandem repeat (STR) profiling to exclude the possibility of cross-contamination with other known cell lines. All cell samples were tested for mycoplasma contamination via polymerase chain reaction (PCR) and showed negative results (Supplementary Fig. 1). L02 cells were cultured in RPMI-1640 medium (Gibco, Carlsbad, CA, USA), while HepG2, PLC/PRF/5, Hep3B, and Huh-7 cells were all cultured in Dulbecco’s modified Eagle medium (DMEM; Gibco) containing 1% streptomycin-penicillin and 10% fetal bovine serum (FBS; Gibco) at 37 °C with 5% CO_2_.

### HCC cell grouping and transfection

(1) Normal L02 cells and HCC cells Hep3B, PLC/PRF/5, HepG2, and Huh-7 were seeded onto 12-well plates (1 × 10^6^ cells/well) and cultured for 48 h. The mRNA and protein expression levels of PFKFB4 were subsequently assessed by real-time quantitative polymerase chain reaction (RT-qPCR) and western blot assays, respectively.

(2) Huh-7 and Hep3B cells were seeded onto 96-well plates (1 × 10^4^ cells/well), separately, which were processed as thereinafter: (1) the Blank group: HCC cells normally cultured for 72 h; (2) the si-NC and si-PFKFB4 groups: HCC cells were transfected with the control plasmid (si-NC) and the PFKFB4 interference plasmid (si-PFKFB4); (3) the oe-NC and oe-PFKFB4 groups: HCC cells were transfected with control plasmid (oe-NC) and PFKFB4 overexpression plasmid (oe-PFKFB4), respectively; (4) oe-PFKFB4 + 2-DG group: HCC cells were transfected with oe-PFKFB4 and subsequently treated with 10 mM 2-deoxy-D-glucose (2-DG; HY-13966, MCE, Monmouth Junction, NJ, USA) for 24 h, as previously described [29]. After 48-h transfection, the transfection efficiency was tested using RT-qPCR and western blot assays. The transfected HCC cells were then incubated for 72 h. The transfection assays were implemented as follows: upon 80% confluence, the cells were subjected to transfection assays using a Lipofectamine™ 2000 Transfection Reagent (11668500, Invitrogen™, Carlsbad, CA, USA). The cells were transfected with si-NC, si-PFKFB4, oe-NC, and oe-PFKFB4 plasmids, respectively, at a final concentration of 200 ng/well. All the plasmids were designed and synthesized by GenePharma (Suzhou, Jiangsu, China).

### Detection of glycolytic indexes

Differently-treated Huh-7 and Hep3B cells were collected for the assessment of glycolysis activity. Glucose uptake, adenosine triphosphate (ATP) production, and lactate production levels were measured by a glucose uptake colorimetric assay kit (MAK083, Sigma-Aldrich, St. Louis, MO, USA), ATP kit (MAK190, Sigma-Aldrich), and lactate kit (MAK329, Sigma-Aldrich) for assessment of glycolysis levels in accordance with the instructions of the kits. All vlaues were normalized to the Blank group and are presented as mean values.

### CCK-8 assay

Cell viability was assessed using a CCK-8 kit (C0037, Beyotime). Differently treated HCC cells were seeded into 96-well plates (1 × 10^4^/well) and incubated for 0, 24, 48, and 72 h at 37 °C with 5% CO_2_. At each time point, 10 µL of CCK-8 solution was added to each well and incubated for another 2 h. A microplate reader (Thermo Scientific, Waltham, MA, USA) was employed for detecting the optical density at 450 nm.

### Transwell migration assay

The migration of HCC cells was evaluated using Transwell assay. Briefly, 600 µL of complete medium comprising 10% FBS was added to the basolateral chamber (3413, Corning, NY, USA), while 200 µL of cell suspension (1 × 10^4^ cells) was seeded into the apical chamber. After 24 h of incubation, non-migrated cells were removed, and migrated cells were fixed with 4% PFA for 30 min and stained with 0.1% crystal violet solution (V5265, Sigma-Aldrich) for 20 min. Migrated cells were visualized and counted under a light microscope (Leica).

### Differentiation and culture of M0 macrophages

Human monocytic THP-1 cells (SNL-044) were obtained from Wuhan Sunn Cell Biotechnology Co., Ltd. and authenticated by STR profiling. Furthermore, THP-1 cells were tested for mycoplasma contamination via PCR and showed negative results (Supplementary Fig. 1). In the light of a previous literature [30], THP-1 cells were differnetiated into M0 macrophages by treatment with 150 nM phorbol myristate acetate (PMA; P8139, Sigma-Aldrich) for 48 h. Mature M0 macrophages were subsequently cultured in DMEM (Gibco) containing 1% streptomycin-penicillin (Gibco) and 10% FBS at 37 °C with 5% CO_2_.

### Co-culture of M0 macrophages and HCC cells and transfection experiments

As previously described [31], the cells were co-cultured in a Transwell co-culture system separated in the middle using a polyester membrane with a pore size of 0.4 μm. To simulate the interactions between TAMs and HCC cells in the TME in vitro, 1 × 10^5^ HCC cells were seeded in the apical chamber, whilst 1 × 10^5^ M0 macrophages were seeded in the basolateral chamber. The grouping was executed as below: (1) the M0 + Co (He-Blank) group and M0 + Co (Hu-Blank) group (M0 macrophages co-cultured with HCC cells [Hep3B and Huh-7 cells] in the Blank group for 72 h); (2) the M0 + Co (He-si-NC), M0 + Co (He-si-PFKFB4), M0 + Co (Hu-si-NC), and M0 + Co (Hu-si-PFKFB4) groups (M0 macrophages co-cultured with si-NC or si-PFKFB4-transfected HCC cells [Hep3B and Huh-7 cells] for 72 h); (3) the M0 + Co (He-oe-NC), M0 + Co (He-oe-PFKFB4), M0 + Co (Hu-oe-NC), and M0 + Co (Hu-oe-PFKFB4) groups (M0 macrophages co-cultured with oe-NC or oe-PFKFB4-transfected HCC cells [Hep3B and Huh-7 cells] for 72 h); (4) the M0 (si-NC) + Co (He-oe-PFKFB4), M0 (si-MCT1) + Co (He-oe-PFKFB4), M0 (si-NC) + Co (Hu-oe-PFKFB4), and M0 (si-MCT1) + Co (Hu-oe-PFKFB4) groups (M0 macrophages transfected with an interfering plasmid for MTC-1 [si-MCT1] and si-NC, and then co-cultured with oe-PFKFB4-transfected HCC cells [Hep3B and Huh-7 cells] for 72 h); (5) the Vehicle + M0 + Co (He-oe-PFKFB4), NaHCO_3_ + M0 + Co (He-oe-PFKFB4), Vehicle + M0 + Co (Hu-oe-PFKFB4), and NaHCO_3_ + M0 + Co (Hu-oe-PFKFB4) groups (the co-culture system of M0 macrophages and oe-PFKFB4-transfected HCC cells [Hep3B and Huh-7 cells] supplemented with 5 mM NaHCO_3_ [32] and solvent [distilled water], respectively, to neutralise lactate produced by glycolysis of HCC cells, and then cultured for 72 h); and (6) the DMSO + M0 + Co (He-oe-PFKFB4), GSK2837808A + M0 + Co (He-oe-PFKFB4), DMSO + M0 + Co (Hu-oe-PFKFB4), and GSK2837808A + M0 + Co (Hu-oe-PFKFB4) groups (M0 macrophages were co-cultured with oe-PFKFB4-transfected HCC cells [Hep3B and Huh-7]. The co-culture systems were treated with 75 µM GSK2837808A [HY-100681, MCE] [33] or dimethyl sulfoxide [DMSO] for 72 h).

### Immunofluorescence staining

The percentage of Arg-1–positive cells (%) was detected in TAMs by immunofluorescence staining. Briefly, M0 macrophages cultured on coverslips were fixed with 4% PFA (P0099-100 mL, Beyotime) for 10 min at 4°C, permabilized, with 0.5% Triton X-100 (GL1659-NEC, Biolab Technology Co., Ltd., Beijing, China) for 15 min, and blocked with 3% BSA (YM-MY563J, Yuanmu Bio-Technology Co., Ltd., Shanghai, China) at room temperature for 30 min. Next, the samples were supplemented with a primary antibody anti-Arg-1 (1/200, ab96183, Abcam) for incubation overnight at 4°C, rinsed with PBS, and cultured at room temperature with an Alexa Fluor^®^ 594 (orange)-labelled IgG H&L secondary antibody (2 µg/mL, ab150092, Abcam) away from light for 1 h. Cell nuclei (blue) were stained with 4’,6-diamidino-2-phenylindole staining solution (ab228549, Abcam). Sections were sealed using an anti-fluorescence quenching sealing agent (A598329, Aladdin Biochemical Technology Co., Ltd., Shanghai, China) and then photographed under a fluorescence microscope (OLYMPUS, Tokyo, Japan). The Image J software (National Institutes of Health) was used to analyze the positive expression rate (%) of the M2 polarization marker Arg-1 in TAMs (M0 macrophages after co-culture). The results were depicted as mean values.

### ELISA

Culture supernatant was collected from the differently-treated TAMs (M0 macrophages after co-culture). The levels of M2 polarization-associated cytokines interleukin (IL)-10 (H009-1-2), IL-4 (H005-1-2), IL-13 (H011), and chemokine ligand 2 (CCL2) (H318-1) were determined, separately, using ELISA kits, which were acquired from Jiancheng Bioengineering Institute (Nanjing, Jiangsu, China). The experiments were conducted on the basis of the instructions.

### Chromatin immunoprecipitation (ChIP) assay

ChIP assay was used to determine the enrichment of H3K18la on M2 polarization-related genes Arg-1 and CD206. The procedures were as follows: co-cultured TAMs (1 × 10^7^ cells) were seeded in dishes (10 cm in diameter), and then crosslinked using 1% formaldehyde for 10 min at 37 °C. The crosslinking reaction was stopped using 0.125 M glycine, with the dishes left for 5 min at room temperature. Chromatin was fragmented into 200–500 nt fragments using sonication. Thereafter, the samples were added with 2 µg of antibodies anti-H3K18la (PTM-1406, PTM BIO, Hangzhou, Zhejiang, China) and anti-IgG (ab6757, Abcam), followed by incubation overnight at 4 °C. After another 6-h incubation with protein A + protein G magnetic beads (16–663, Millipore, Billerica, MA, USA), proteins were pulled down at 4 °C. Following crosslinking reversal and proteinase K (1.24568, Sigma-Aldrich) treatment, DNA was released from bound chromatin, then precipitated, and diluted with 100 µL of 0.2 M glycine, to construct purified DNA fragments. Finally, enrichment levels of H3K18la in the promoter region of Arg-1 and CD206 were assayed by qPCR.

### Xenograft tumor experiment in nude mice

Specific-pathogen free grade BALB/c nude mice (4–6 weeks old, Lanzhou Institute of Biological Products Co., Ltd., Lanzhou, Gansu, China) were treated as hereinafter: (1) the Control group (*n* = 12): (during the logarithmic growth period, Huh-7 cells [100 µL, 5 × 10^6^ cells] were inoculated into the axilla of the nude mice for the establishment of a xenograft tumor model); (2) the Lv-NC (*n* = 12), Lv-oe-PFKFB4 (*n* = 12) and Lv-si-PFKFB4 (*n* = 12) groups: (negative control (Lv-NC), PFKFB4 overexpression (Lv-oe-PFKFB4) and PFKFB4 interfering (Lv-si-PFKFB4) lentiviral-transfected Huh-7 cells were inoculated into the axillae of nude mice, respectively, for the establishment of a xenograft tumor model). The measurement of the xenograft tumor volume was implemented once a week (tumor volume = length × width^2^ × 0.5). On the 35th day, the tumor volume was examined for the last time. Then, mice were injected intraperitoneally with an overdose of pentobarbital sodium (3%, 100 mg/kg) for euthanasia, and subsequently, the tumors were subjected to observation, photographing, and weighing. A portion of tumor tissues was applied for assessing the mRNA and protein levels of PFKFB4 by RT-qPCR and western blot assays, measuring the protein levels of Arg-1, H3K18la, and CD206, and determining the levels of lactate in the tumor tissues using kits. The detection method of lactate was consistent with the cellular experiments. The percentage of TAM M2-polarized cells was finally assayed in the remaining tumor tissues using flow cytometry.

### RT-qPCR experiments

Total RNA was isolated from HCC cells, TAMs, or nude mouse tumor tissues by total RNA extraction reagent (YM-0110-168, Yuanmu Bio-Technology Co., Ltd.), whose concentration and purification were tested using Nanodrop2000 micro UV spectrophotometer (Thermo Scientific). In short, complementary DNA was reverse transcribed from RNA by a reverse transcription kit (FSQ-101, TOYOBO, Osaka, Japan). RT-qPCR was then performed on a 7900 HT real-time PCR system (Applied Biosystems, Foster City, CA, USA) using a DyNAmo HS SYBR Green qPCR kit (F410L, Thermo Scientific). With β-actin acting as the internal parameter, the 2^−ΔΔCt^ method was used for the calculation of relative expression levels of target genes. The data of the control group underwent normalization, with the final outcomes represented as mean values. PCR primer sequences are illustrated in Table 1.

**Table 1:** RT-qPCR primer sequences

Gene	Forward 5’-3’	Reverse 5’-3’
PFKFB4	GGAGTTCAATGTTGGCCAGT	TCAGGATCCACACAGATGGA
Arg-1	GGTGTTGCCTGCTGCCTTCC	GTTCTGAAGAGGTGAGTGGCTGTC
CD206	GAGCAAACATACCTGACAGGATTA	GGACTTCCTGGTAACCAGTTCA
MCT1	TGTTGTTGCAAATGGAGTGT	AAGTCGATAATTGATGCCCATGCCAA
β-actin	TTCCTTCCTGGGCATGGAGT	TACAGGTCTTTGCGGATGTC

### Western blot assay

Total proteins were extracted from nude mouse tumor tissues, HCC cells, or TAMs, using RIPA lysate (YM-S2010, Yuanmu Bio-Technology Co., Ltd.), whose concentration was tested by a bicinchoninic acid protein assay kit (PA115-01, Tiangen Biotech, Beijing, China). The proteins (20 µg) were loaded in each well. After that, the samples were separated by 10% sodium dodecyl sulfate-polyacrylamide gel electrophoresis and transferred to a polyvinylidene fluoride membrane, which was then blocked with 5% skim milk for 2 h. Following this, the samples were added with primary antibodies for incubation overnight at 4 °C, including anti-PFKFB4 (1/1000, ab137785, Abcam), anti-CD206 (1/1000, ab64693, Abcam), anti-Arg-1 (1/2000, ab96183, Abcam), anti-iNOS (1/2000, ab178945, Abcam), anti-CD86 (1/1000, ab239075, Abcam), anti-PanKla (1/1000, PTM-1401, PTM BIO), anti-MCT1 (1/1000, ab314172, Abcam), anti-H3K18la (1/1000, PTM-1406, PTM BIO), anti-Histone H3 (1/1000, ab1791, Abcam) and anti-β-actin (1/1000, ab8227, Abcam). Subsequent to sufficient washes with Tris-buffered saline with Tween-20, the samples were added and incubated with HRP-coupled IgG H&L secondary antibody (1/5000, ab6721, Abcam) at room temperature for 1 h, with images visualized by electrochemiluminescence and captured. The Image J software (National Institutes of Health) was employed for analyzing the band density values, with β-actin and Histone H3 serving as the internal references for total proteins and histones, respectively.

### Flow cytometry

Clinical HCC tissues or HCC xenograft tumor tissues were cut into pieces, and then incubated for 2 h at 37 °C with dissociation solution (containing 7 mL of DMEM + Liberase DH detachment enzyme + 10% FBS + 0.004% DNase I + 0.02% hyaluronidase) (Liberase DH digestion enzymes, DNase I, and hyaluronidase were all purchased from Roche [Basel, Switzerland]). The detached tumor tissue cells were filtered using a 70 μm sterile filter (BD, Franklin Lakes, NJ, USA), subjected to a 5-min centrifugation at 500 × g and 4 °C, and resuspended in PBS to generate a single-cell suspension. Single cell suspensions were adjusted to 1 × 10^6^ cells/mL and resuspended in 200 µL of PBS solution. After that, clinical HCC tissue-derived cells were supplemented with anti-human flow-type primary antibodies, such as FITC Anti-CD68 (11-0689-42, eBioscience™, San Diego, CA, USA), PE/Cy7 anti-CD206 (ab270682, Abcam), and PE anti-CD86 (12-0869-42, eBioscience™). Additionally, cells derived from nude mouse xenograft tumors were added with anti-mouse flow-type primary antibodies, comprising PE/Cy7 anti-CD206 (ab270682, Abcam) and FITC anti-F4/80 (11-4801-82, eBioscience™). Cells were cultured away from light at 4 °C for 30 min, rinsed with PBS, and resuspended in 200 µL of PBS solution. The infiltration percentages (%) of M1-polarized TAMs with CD68^+^CD86^+^ and those with CD68^+^CD206^+^ phenotype in the clinical HCC tissues, as well as the infiltration percentage (%)of M2-polarized TAMs with F4/80^+^CD206^+^ in the xenograft tumors in nude mice, were analysed on a flow cytometer (FACS Canto II, BD).

### Statistical analysis

Data statistical analysis and graphing were conducted using SPSS 22.0 statistical software (IBM Corp., Armonk, NY, USA) and GraphPad Prism 9.0 software (GraphPad Software, San Diego, CA, USA). The correlations between the positive expression rate of PFKFB4 in clinical HCC samples and the infiltration percentages of TAM M1 and M2 phenotypes were analyzed by the Pearson correlation coefficient method. All cellular experiments were performed in triplicate, with the sample size of each group in animal experiments set as *n* = 6. The measurement data were tested to be normally distributed by the Shapiro-Wilk test, and were described as mean ± standard deviation. The *t*-test was adopted for comparisons between two groups, while one-way analysis of variance (ANOVA) was used for comparisons among multiple groups, with Tukey’s test used for post hoc analysis. The differences were deemed to be statistically significant at *P* < 0.05.

## Results

### Glycolysis-related gene PFKFB4 was up-regulated in HCC, and associated with poor prognosis

**Fig. 1:**
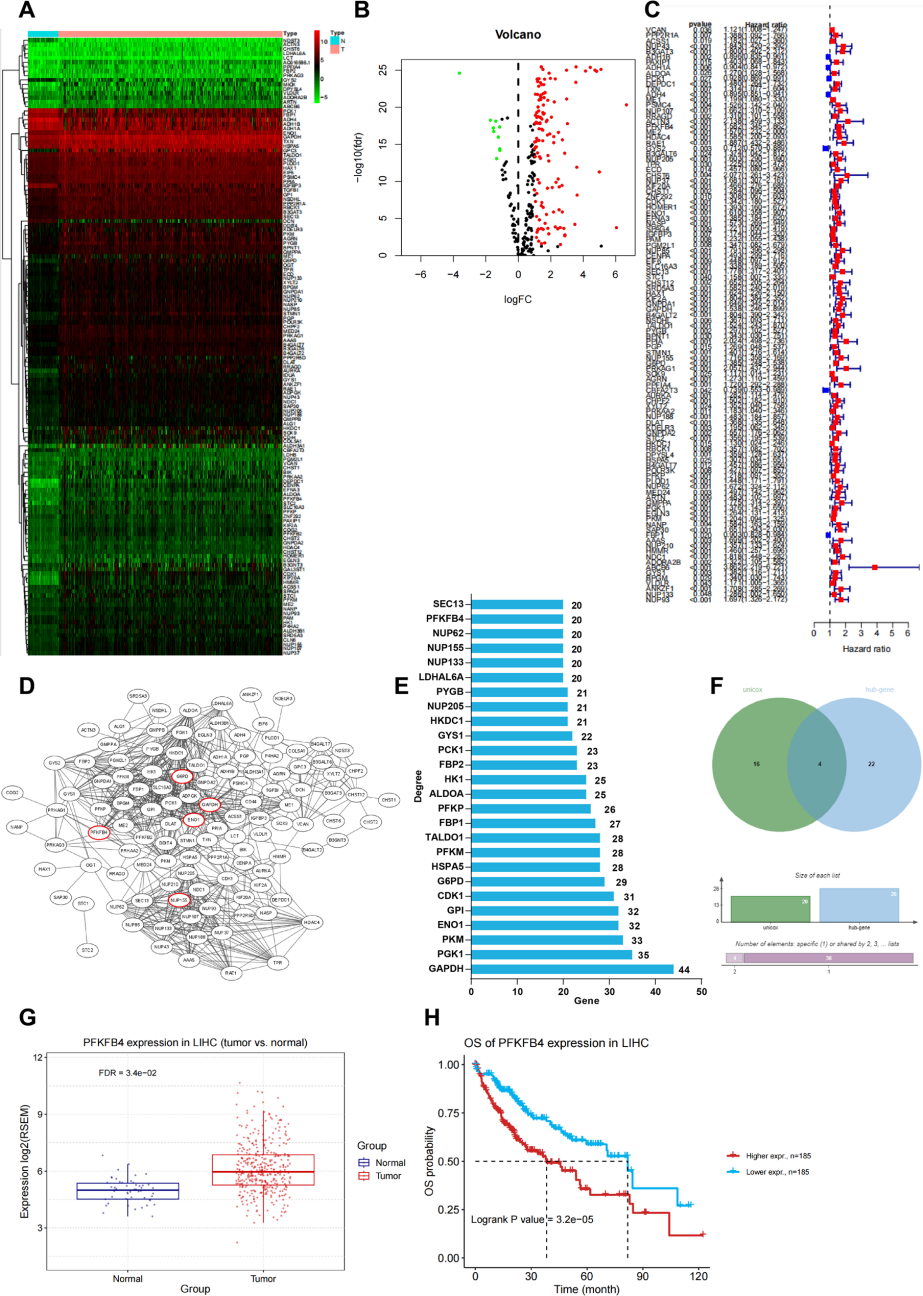
Glycolysis-related gene PFKFB4 had up-regulated expression in HCC, which was associated with the poor prognosis. The glycolytic pathway and HCC sample datasets were respectively downloaded from the GSEA database (https://www.gsea-msigdb.org/gsea/index.jsp) and TCGA GDC database (https://portal.gdc.cancer.gov/). The differential expression levels of glycolysis-related genes in HCC were analyzed by R language. **A**: Heatmap of glycolytic gene differential expression patterns in HCC samples; **B**: Volcano plot of glycolytic gene differential expression patterns in HCC, with |logFC| > 1 and FDR < 0.05 as the screening criteria for differentially-expressed genes; **C**: Univariate Cox analysis of the differentially-expressed glycolytic genes and survival of HCC cases; D: The STRING database (https://cn.string-db.org/) was used to plot the protein interaction map for differentially-expressed glycolytic genes, where the connecting line between two genes in the map represented the presence of interaction between them. The more connecting lines the genes had, the larger the degree value was, and the more core position they were in the network graph; **E**: The statistics of degree values of the top 15 genes with the highest core. The horizontal coordinate in the graph indicated the degree value, and the vertical coordinate indicated the gene name; **F**: The intersection of the two data sets of the top 20 genes with the most significant survival associations and the genes with a degree value over 20 in the univariate Cox analysis, which was indicated by the middle part of the graph; **G**: Scatter plot of PFKFB4 expression in normal and HCC samples analyzed by bioinformatics database GSCA (http://bioinfo.life.hust.edu.cn/GSCA/#/); **H**: Kaplan-Meier survival curve to analyze the relationship between PFKFB4 expression and prognosis in patients with HCC

The datasets of glycolytic pathway and HCC samples were downloaded from TCGA GDC and GSEA databases. R language was utilised to analyze the differential expression of glycolysis-related genes in HCC, and identified 140 differentially-expressed glycolysis-related genes (Fig. 1A-B, all *P* < 0.05). Univariate Cox regression analysis identified a significant association between 108 genes and the overall survival in HCC patients (Fig. 1C, all *P* < 0.05). Protein-protein interaction of these 140 genes were analysed, and a protein interaction network diagram was set up, followed by the calculation of the degree value of each gene in the network diagram. The findings manifested that several genes, including GAPDH, G6PD, PFKFB4, ENO1, and NUP155, occupied important positions within the network network (Fig. 1D-E). Intersection analysis between genes with a degree value > 20 and the top 20 prognostically significant genes identified four candidate genes: PFKFB4, G6PD, NUP155, and ENO1 (Fig. 1F). Given previous reports implicating PFKFB4 in HCC glycolysis and tumor progression [34, 35], PFKFB4 was selected as the target gene in the study.

Analysis using the Gene Set Cancer Analysis (https://guolab.wchscu.cn/GSCA/#/) further confirmed that PFKFB4 expression was significantly elevated in HCC tissues (Fig. 1G, *P* < 0.05), that high expression of PFKFB4 was associated with poor prognosis in HCC patients (Fig. 1H, *P* < 0.05).

### PFKFB4 expression was elevated in HCC tissues and correlated with TAM infiltration and polarization

**Fig. 2:**
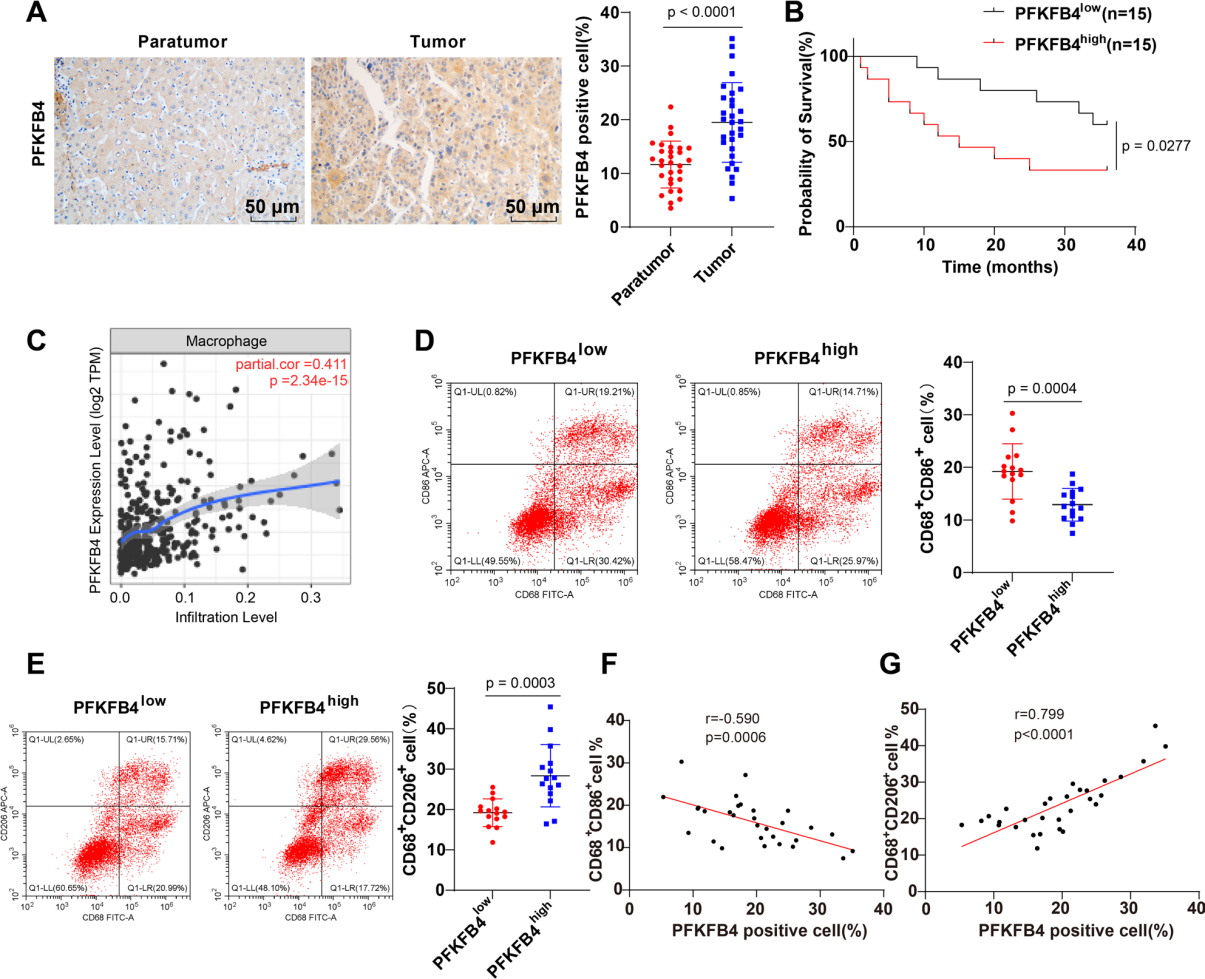
The expression of PFKFB4 ascended in HCC clinical tissues, and had relevance to the infiltration of TAMs. HCC tissues and adjacent tissues were harvested from 30 HCC cases and analyzed for (**A**) The positive expression rate (%) of PFKFB4 by immunohistochemistry; **B**: The relationship between the expression of PFKFB4 and the prognosis in HCC cases by Kaplan-Meier survival curve; **C**: TIMER database analysis of the relationship between PFKFB4 expression and macrophage infiltration; **D**-**E**: The infiltration percentage (%) of M1-polarized (CD68^+^CD86^+^) TAMs (panel **D**) and M2-polarized (CD68^+^CD206^+^) TAMs (panel **E**) in HCC tissues by flow cytometry; **F**-**G**: The correlations between the positive expression rate (%) of PFKFB4 and the infiltration percentages (%) of (panel **F**) M1- and (panel **G**) M2-polarized TAMs in HCC tissues by Pearson correlation coefficient method. Comparisons between two groups were carried out using a *t*-test. *P* < 0.05 indicated that the difference was statistically significant

To further ascertain the role of PFKFB4 in HCC, tumor tissues and adjacent non-tumor tissues were collected from 30 HCC patients. Clinical baseline data of the patients were also collected, including sex, age, BMI, smoking history, drinking history, creatinine, ALT, AST, ALP, GGT, albumin, AFP, tumor size, TNM stage, and recurrence status (see Supplementary Table 1). As indicated by IHC detection, the positive expression rate of PFKFB4 was elevated in HCC tissues (Fig. 2A, *P* < 0.05).

The 30 HCC patients were subsequently allocated into the PFKFB4^high^ and PFKFB4^low^ groups based on PFKFB4 expression levels. Further analysis was performed to compare PFKFB4 levels with the clinical characteristics of HCC patients (Table 2). Significant differences were observed between the PFKFB4^low^ and PFKFB4^high^ groups in terms of ALT, AST, ALP, GGT, albumin, AFP, tumor size, and TNM stage (all *P* < 0.05). In contrast, no statistically significant differences were found in sex, age, BMI, smoking history, alcohol consumption history, creatinine, or recurrence status (all *P* > 0.05) (Table 2). Kaplan-Meier survival analysis demonstrated that patients in PFKFB4^high^ group exhibited significantly poorer overall survival (Fig. 2B, *P* < 0.05).

**Table 2:** The correlation between PFKFB4 levels and clinical characteristics of HCC patients

Items		PFKFB4^low^ (*n* = 15)	PFKFB4^high^ (*n* = 15)	*P* value
Age (years)		59.3 ± 5.6	58.0 ± 5.3	0.5150
Sex [n, (%)]		12 (80.00)	10 (66.67)	0.6817
Smoking history [n, (%)]		2 (13.33)	3 (20.00)	> 0.9999
Drinking history [n, (%)]		2 (13.33)	2 (13.33)	> 0.9999
BMI		25.0 ± 3.8	25.6 ± 3.5	0.7068
Creatinine (mg/dL)		0.9 ± 0.2	1.0 ± 0.2	0.0762
ALT (U/L)		59.2 ± 21.5	98.7 ± 26.2	0.0001
AST (U/L)		62.3 ± 320.5	101.9 ± 24.7	0.0001
ALP (U/L)		117.1 ± 29.9	153.2 ± 39.8	0.0090
GGT (U/L)		74.6 ± 33.4	123.7 ± 39.2	0.0009
Albumin (g/dL)		4.9 ± 0.6	4.2 ± 0.6	0.0033
AFP (ng/mL)	≤ 400	1 (6.67)	9 (60.00)	0.005
	> 400	14 (93.33)	6 (40.00)	
Tumor size (cm)	≤ 5	12 (80.00)	5 (33.33)	0.0253
	> 5	3 (20.00)	10 (66.67)	
TNM stage [n, (%)]	I	9 (60.00)	3 (20.00)	0.0369
	II	4 (26.67)	4 (26.67)	
	III	2 (13.33)	8 (53.33)	0.0656
	IV	0 (0.00)	0 (0.00)	
Recurrence [n, (%)]		5 (33.33)	11 (73.33)	

Note: BMI, body mass index; AST, aspartate transaminase; ALT, alanine transaminase; ALP, alkaline phosphatase; GGT, gamma-glutamyl transferase; AFP, alpha-fetoprotein. Enumeration data were presented as cases and percentages and were compared using the chi-square test. Measurement data conforming to a normal distribution were expressed as mean ± standard deviation. Comparisons between groups were conducted using the independent samples *t*-test. A *P* value < 0.05 was considered statistically significant

Moreover, analysis via the TIMER database revealed a positive correlation between PFKFB4 expression and macrophage infiltration in HCC (Fig. 2C). Additionally, research has suggested that M2-type macrophages can promote the malignant progression of HCC [36]. The percentages of M1-polarized TAMs (CD68^+^CD86^+^) and M2-polarized TAMs (CD68^+^CD206^+^) in HCC tumor tissues were examined by flow cytometry, with the results showing a decrease in the percentage of M1-polarized TAMs (Fig. 2D, *P* < 0.05) and an increase in the percentage of M2-polarized TAMs (Fig. 2E, *P* < 0.05) in the PFKFB4^high^ group in comparison with the PFKFB4^low^ group.

In addition, Pearson correlation coefficient method found that PFKFB4 negatively correlated with M1 TAM infiltration (Fig. 2F, *r* = -0.590, *P* < 0.05), while positively correlated with M2 TAM infiltration (Fig. 2G, *r* = 0.799, *P* < 0.05), suggesting a potential role of PFKFB4 promoting M2 polarization of TAMs in HCC tissues.

### PFKFB4 mediated lactate production via aerobic glycolysis, and enhanced HCC cell migration and proliferation

**Fig. 3:**
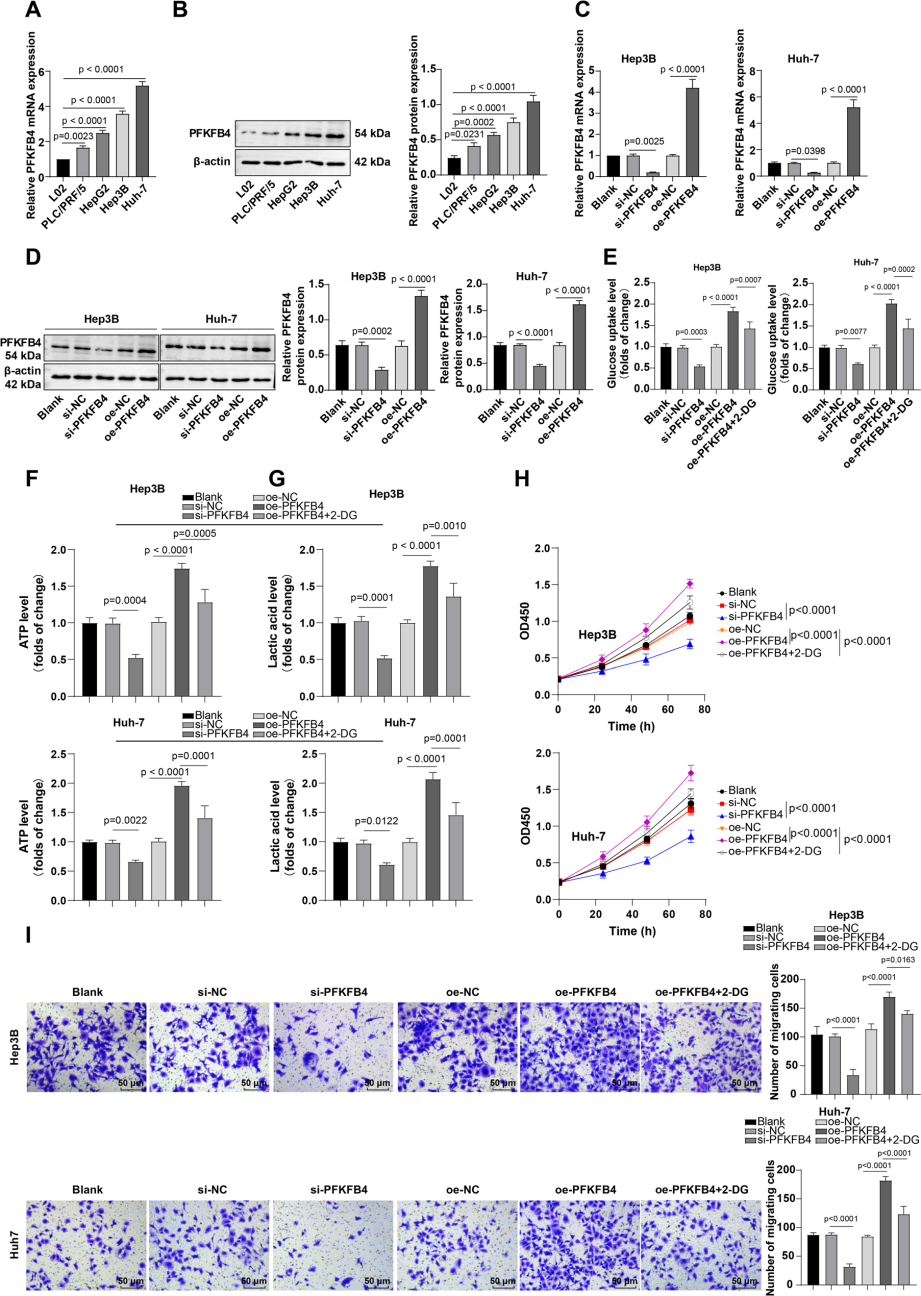
PFKFB4 mediated lactate production via aerobic glycolysis, and contributed to HCC cell migration and proliferation. A-B: RT-qPCR and western blot assays to assess the cellular mRNA (**A**) and protein (**B**) expression patterns of PFKFB4. Hep3B and Huh-7 were transduced respectively with overexpression plasmid (oe-PFKFB4) and interfering RNA plasmid (si-PFKFB4) of PFKFB4 to increase or decrease the PFKFB4 expression; **C**-**D**: The mRNA (**C**) and protein (**D**) expression levels of PFKFB4 in HCC cells assessed by RT-qPCR and western blot; **E**-**G**: Determination of the glucose uptake (**E**), ATP production (**F**), and lactic acid production (**G**) levels in HCC cells using the kits; **H**-**I**: The assessment of proliferative and migratory abilities in HCC cells via CCK-8 (**H**) and Transwell (**I**) assays. Three repetitions were guaranteed in cell experiments independently, with the data signified as the mean ± standard deviation. Data comparisons among multiple groups were executed using one-way ANOVA. *P* < 0.05 indicated that the difference was statistically significant

HCC cell lines (PLC/PRF/5, HepG2, Hep3B, and Huh-7) and normal hepatocytes (L02) were cultured in vitro. RT-qPCR and western blot assays disclosed upregulations in the levels of PFKFB4 in the four HCC cell lines (Fig. 3A-B, all *P* < 0.05). Among them, Huh-7 and Hep3B cells, which exhibited relatively high PFKFB4 expression, were selected for subsequent experiments.

To explore whether PFKFB4 modulates HCC cell migration and proliferation through aerobic glycolysis, interfering RNA (si-PFKFB4) and overexpression (oe-PFKFB4) plasmids of PFKFB4 were transfected into Hep3B and Huh-7 for down-regulating or up-regulating the expression of PFKFB4. RT-qPCR and western blot assays indicated that PFKFB4 expression in HCC cells was down-regulated or up-regulated by si-PFKFB4 or oe-PFKFB4, respectively (Fig. 3C-D, all *P* < 0.05).

Moreover, the kit detection found that the levels of glucose uptake, ATP production, and lactate production in HCC cells were diminished by si-PFKFB4 treatment, whereas these levels were heightened after oe-PFKFB4 treatment (Fig. 3E-G, all *P* < 0.05). These results indicated that PFKFB4 promotes aerobic glycolysis in HCC cells. However, after transfection with oe-PFKFB4 and treatment with the glycolytic inhibitor 2-DG, the levels of glucose uptake, ATP production, and lactate generation in HCC cells were all reduced (Fig. 3E-G, all *P* < 0.05). Consistently, CCK-8 and Transwell assays demonstrated that the proliferative and migratory abilities of HCC cells were dampened by si-PFKFB4 treatment, but were strengthened by oe-PFKFB4 treatment; however, following transfection with oe-PFKFB4 and treatment with 2-DG, the proliferative and migratory capacities of HCC cells were reduced (Fig. 3H-I, all *P* < 0.05). These findings collectively demonstrate that PFKFB4 facilitates HCC cell proliferation and migration by mediating lactate production through aerobic glycolysis.

### Alterations in PFKFB4 expression indirectly influenced TAM M2 polarization in HCC cells

On the basis of the previous study, the effect of PFKFB4 on the M2 polarization of TAMs in HCC cells was further explored. THP-1 cells were first differentiated into M0 macrophages by treatment with 150 nM PMA for 48 h as previously described [30]. Immunofluorescence staining revealed a positivity rate exceeding 95% for CD68 expression after PMA-induced differentiation (Supplementary Fig. 2).

**Fig. 4:**
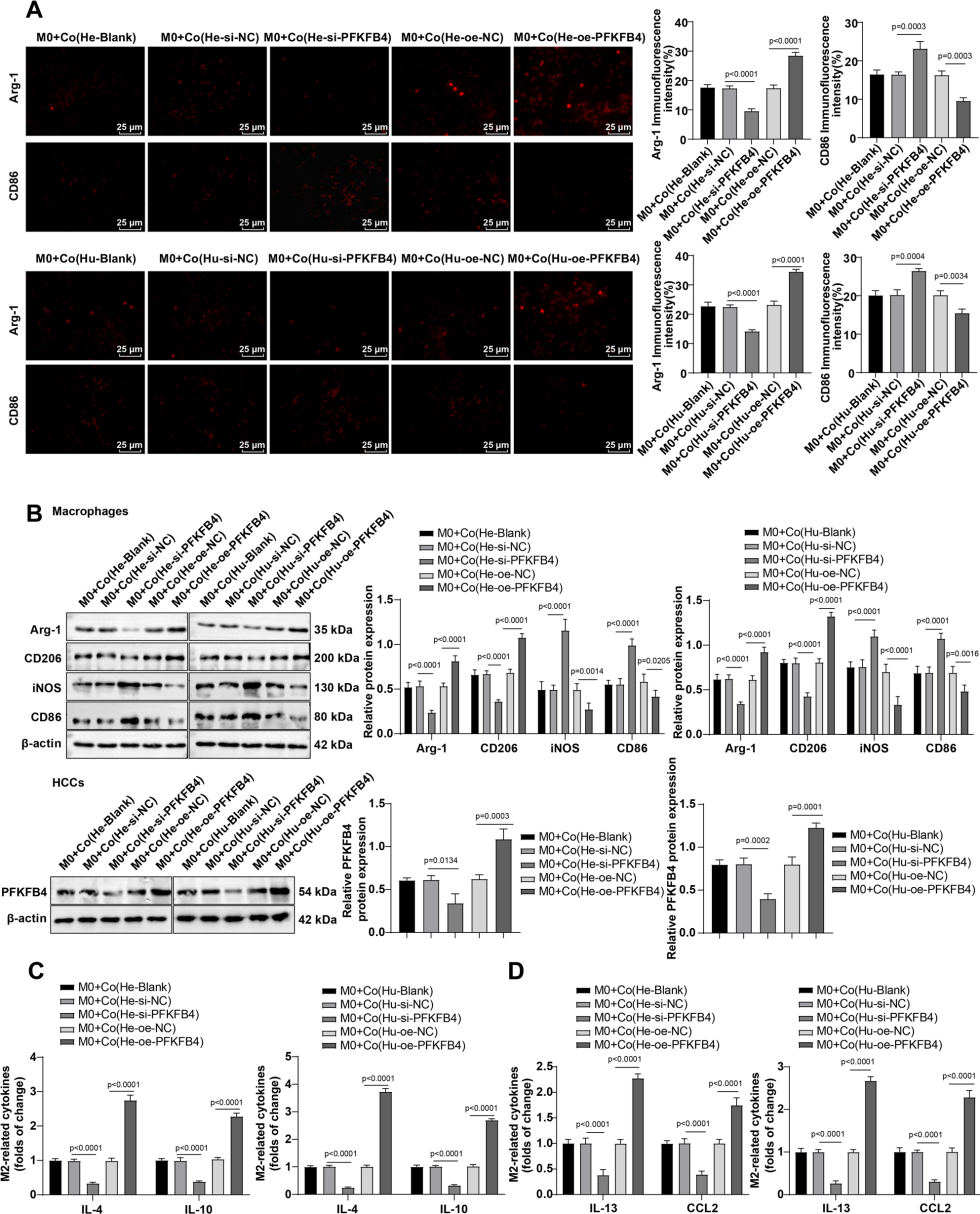
Variations of PFKFB4 expression could indirectly influence M2 polarization of TAMs in HCC cells. The in vitro interaction between TAMs and HCC cells in the TME was simulated by indirectly co-culturing M0 macrophages and differently-treated Hep3B and Huh-7 cells (cells in the Blank group or transduced with si-PFKFB4, oe-PFKFB4, si-NC, and oe-NC) in a Transwell system. **A**: Detection of Arg-1 and CD86 positive expression rate by immunofluorescence staining; **B**: Western blot to test the protein expression levels of PFKFB4 in tumor cells and M2 polarization markers (Arg-1 and CD206) in TAMs; **C**-**D**: ELISA to determine the levels of IL-4, IL-10, IL-13, and CCL2 in the co-culture supernatant. Cell experiments were repeated thrice. Data were presented as mean ± standard deviation, and comparisons among multiple groups were made using one-way ANOVA. *P* < 0.05 indicated that the difference was statistically significant

Subsequently, M0 macrophages were co-cultured indirectly with Hep3B and Huh-7 with different treatments to mimic the interactions between TAMs and HCC cells within the TME. Immunofluorescence staining results revealed that co-culture with si-PFKFB4-treated HCC cells led to decreased PFKFB4 expression in HCC cells, accompanied by reduced Arg-1-positive expression and increased CD86 expression in TAM cells. Conversely, co-culturewith oe-PFKFB4-treated HCC cells resulted in elevated Arg-1-positive expression and decreased CD86expression in TAMs (Fig. 4A, all *P* < 0.05).

Western blot assay indicated that knockdown of PFKFB4 in HCC cells significantly decreased the expression of Arg-1 and CD206 in co-cultured macrophages., whereas overexpression of PFKFB4 produced the opposite effects (Fig. 4B, all *P* < 0.05). Next, ELISA results displayed that after co-culture with PFKFB4-knockdown HCC cells, the levels of IL-4, IL-10, IL-13, and CCL2 in the supernatant of the co-culture system were reduced; however, co-culture with PFKFB4-overexpressing HCC cells led to increased levels of IL-4, IL-10, IL-13, and CCL2 in the co-culture supernatant (Fig. 4C-D, all *P* < 0.05). The aforementioned results suggest that PFKFB4 expression in HCC cells indirectly promotes TAM M2 polarization within the TME.

### PFKFB4 regulated TAM M2 polarization via histone H3K18la lactylation in HCC cells

**Fig. 5:**
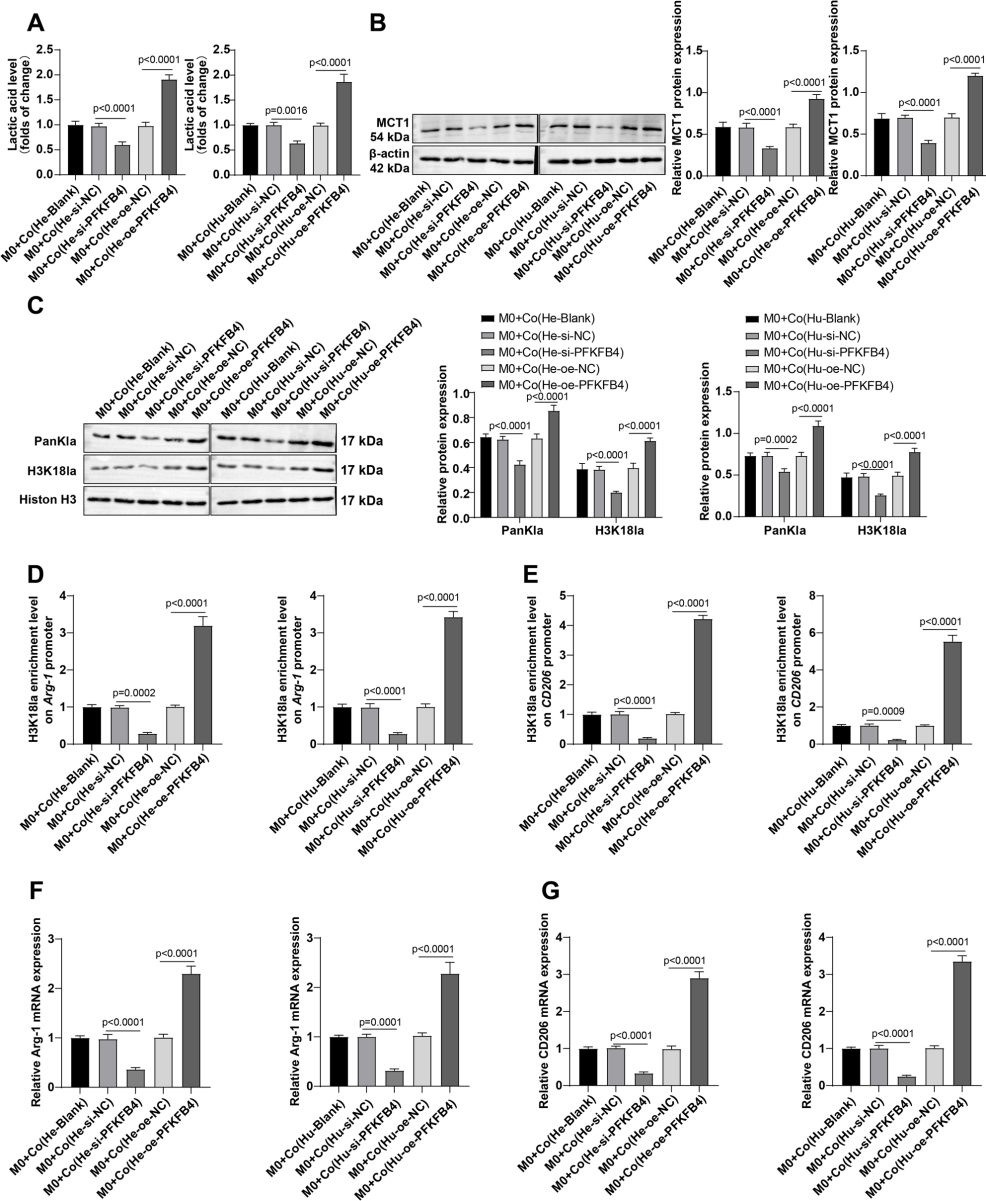
PFKFB4 controlled the M2 polarization-related gene expression patterns in TAMs of HCC cells through lactate/histone H3K18 lactylation. M0 macrophages underwent indirect co-culture with Huh-7 and Hep3B cells with different treatments (cells in the Blank group or transfected with si-NC, oe-NC, oe-PFKFB4, and si-PFKFB4) in the Transwell system. **A**: Measurement of lactate levels in the co-culture supernatant using the kit; B: Western blot for the detection of MCT1 protein expression in TAMs; **C**: Western blot to assess the protein levels of H3K18la and PanKla in TAMs; **D**-**E**: The H3K18la enrichment levels on the Arg-1 and CD206 promoters were assayed by ChIP assay; **F**-**G**: RT-qPCR to determine the Arg-1 and CD206 mRNA transcript levels in TAMs. The cellular experiments were repeated 3 times, with the data depicted as mean ± standard deviation. Comparisons among multiple groups were made using one-way ANOVA. *P* < 0.05 indicated that the difference was statistically significant

Next, we examined whether PFKFB4 in HCC cells promoted lactate accumulation via the glycolytic pathway in the TME, thereby affecting M2 polarization-associated gene expression levels and histone lactylation in TAMs. The lactate levels in cell supernatant and the lactate transporter protein MCT1 levels in TAMs were then examined. Co-culture of macrophages with PFKFB4-knockdown HCC cells resulted in a significant reduction in lactate levels in the supernatant, whereas co-culture with PFKFB4-overexpressing HCC cells markedly increased lactate concentrations (Fig. 5A, all *P* < 0.05). Consistently, MCT1 protein expression in macrophages was decreased following co-culture with PFKFB4-silenced HCC cells, but was elevated after co-culture with PFKFB4-overexpressing HCC cells (Fig. 5B, all *P* < 0.05). These results suggest that PFKFB4-overexpresing HCC cells enhances were capable of stimulating the expression of MCT1 in TAMs and increasing their lactate uptake capacity.

Subsequent detection further discovered that following co-culture of macrophages with PFKFB4-knockdown HCC cells, PanKla and H3K18la levels in TAMs were significantly decreased; however, co-culture with PFKFB4-overexpressing HCC cells resulted in elevated PanKla and H3K18la levels in TAMs (Fig. 5C, all *P* < 0.05). Additionally, after co-culture of macrophages with PFKFB4-knockdown HCC cells, H3K18la enrichment at the promoters of Arg-1 and CD206 in macrophages was reduced, while co-culture with PFKFB4-overexpressing HCC cells increased H3K18la enrichment at the Arg-1 and CD206 promoters in TAMs (Fig. 5D-E, all *P* < 0.05). Changes in mRNA transcript levels of CD206 and Arg-1 were further confirmed that PFKFB4 could modulate the expression patterns of TAM M2 polarization-related genes, CD206 and Arg-1, via H3K18la in HCC cells (Fig. 5F-G, all *P* < 0.05). Collectively, these results indicate that PFKFB4 in HCC cells regulates M2 polarization of TAMs through histone H3K18la.

### Inhibition of lactate uptake in TAMs partially reversed the PFKFB4-induced M2 polarization in HCC cells

**Fig. 6:**
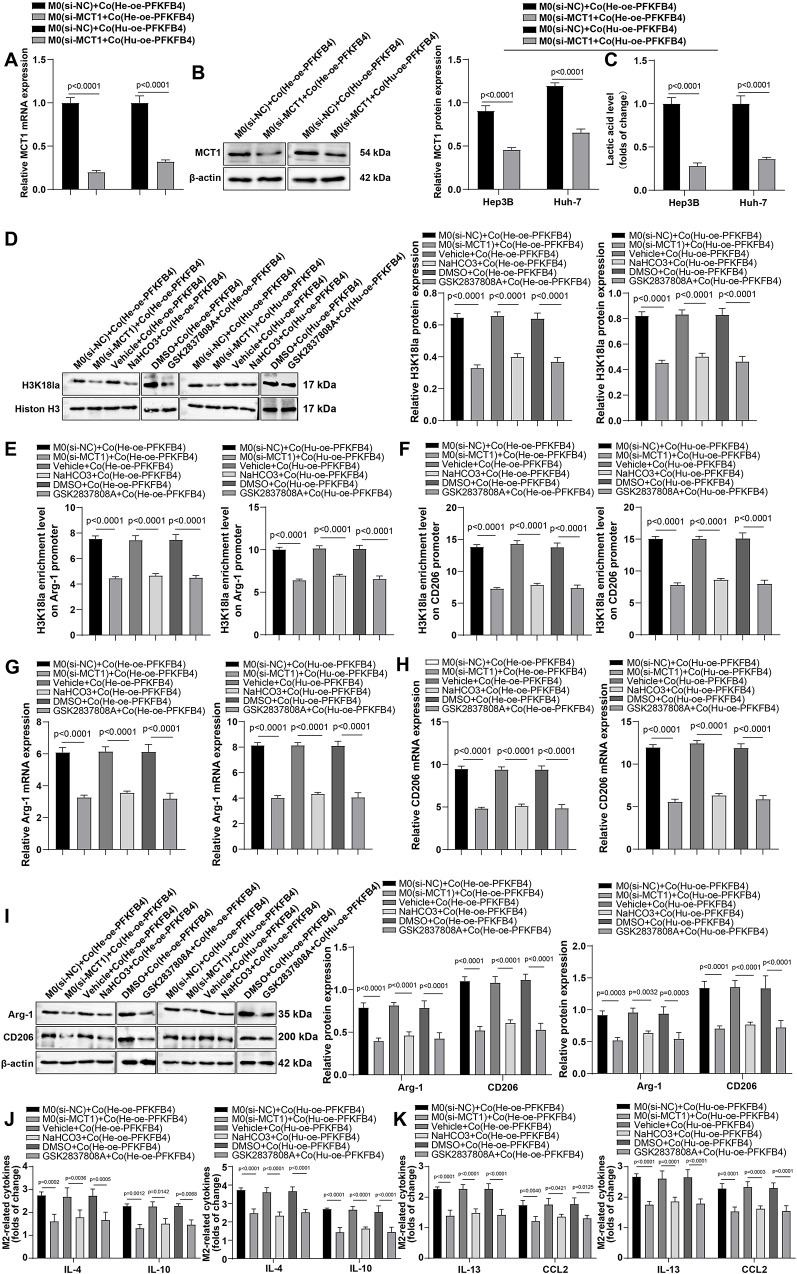
The promotional role of PFKFB4 in M2 polarization of TAMs was partially annulled by the containment of lactate uptake by TAMs in HCC cells. M0 macrophages were transduced with interfering RNA of MCT1 (si-MCT1) for repressing the uptake of lactate by TAMs, or NaHCO_3_ was added to the co-culture supernatant for inhibiting the lactate accumulation in the co-culture system, and M0 macrophages underwent co-culture with oe-PFKFB4-transfected Hep3B and Huh-7 cells. **A**-**B**: Assessment of MCT1 expression levels in TAMs by western blot and RT-qPCR assays; **C**: The kit was applied for testing lactate level in the co-culture supernatant; **D**: The H3K18la level in TAMs was assayed using western blot assay; **E**-**F**: ChIP assay to assess H3K18la enrichment levels on the CD206 and Arg-1 promoters; **G**-**I**: The determination of Arg-1 and CD206 mRNA and protein levels in TAMs by western blot and RT-qPCR; **J**-**K**: ELISA was used for the measurement of IL-4, IL-10, IL-13, and CCL2 levels in the co-culture supernatant. The cellular experiments were repeated three times, and the data were expressed as mean ± standard deviation. Comparisons among multiple groups were made using one-way ANOVA. *P* < 0.05 indicated that the difference was statistically significant

Small interfering RNA targeting MCT1 (si-MCT1) was transfected into M0 macrophages to inhibit lactate uptake by TAMs, or NaHCO_3_ was added to the co-culture supernatant to reduce the lactate accumulation in the co-culture system. RT-qPCR and western blot experiments confirmed that the levels of MCT1 were significantly reduced following si-MCT1 transfection (Fig. 6A-B, all *P* < 0.05). Meanwhile, NaHCO_3_ successfully decreased the level of lactate in the co-culture system (Fig. 6C, all *P* < 0.05). Further treatment of NaHCO_3_, si-MCT1 or the specific lactate dehydrogenase inhibitor GSK2837808A led to decreased H3K18la levels in TAMs (Fig. 6D, all *P* < 0.05). Consistently, H3K18la enrichment levels at the promoters of Arg-1 and CD206 was significantly decreased (Fig. 6E-F, all *P* < 0.05), accompanied by reduced mRNA and protein levels of CD206 and Arg-1 in TAMs (Fig. 6G-I, all *P* < 0.05). In addition, the levels of TAM M2 polarization-related cytokines, including IL-4, IL-10, IL-13, and CCL2 in the co-culture system were significantly decreased (Fig. 6J-K, all *P* < 0.05). This evidence suggests that PFKFB4 in HCC regulates Arg-1 and CD206 expression levels in TAMs via the glycolysis/lactate/H3K18la axis, thereby promoting TAM M2 polarization.

### PFKFB4 stimulated M2 polarization of TAMs and tumor growth through the glycolysis/H3K18la pathway in vivo

**Fig. 7:**
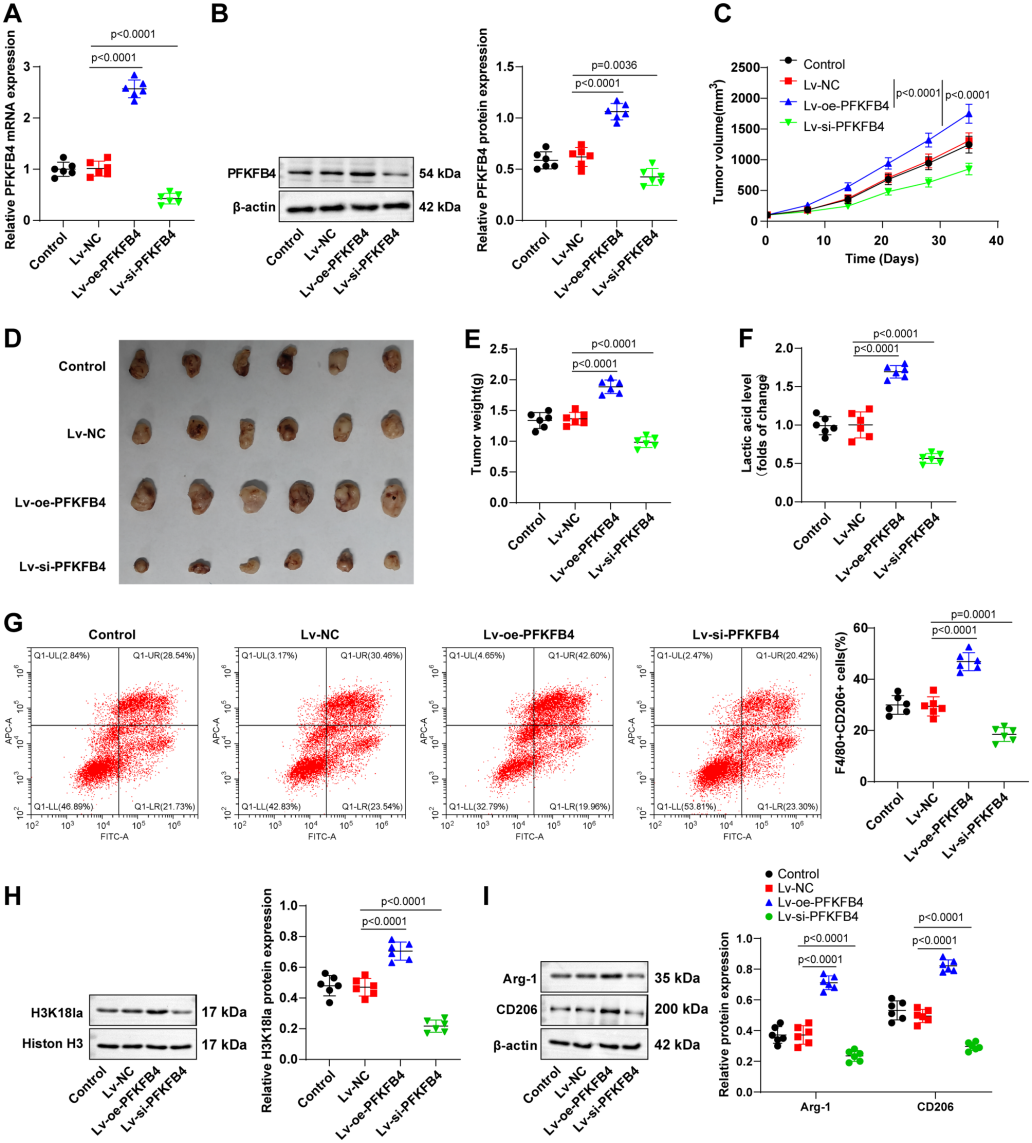
PFKFB4 stimulated M2 polarization of TAMs via the glycolysis/H3K18la pathway, thus accelerating tumor growth in vivo. Huh-7 cells were infected with lentiviruses interfering with PFKFB4 expression (Lv-si-PFKFB4) or overexpressing PFKFB4 (Lv-oe-PFKFB4). Nude mice were then injected with differently-treated Huh-7 cells to build a xenograft tumor model. **A**-**B**: Western blot and RT-qPCR to assess the expression of PFKFB4 in tumor tissues; **C**-**E**: Tumor weight, tumor size and tumor volume changes were observed; F: The kit to determine lactate levels in tumor tissues; **G**: Flow cytometry to detect the percentage of M2 phenotype (F4/80^+^CD206^+^) of TAMs in tumor tissues; **H**: Western blot to detect the level of H3K18la in tumor tissues; **I**: CD206 and Arg-1 expression patterns were tested using western blot. Animal experiments were performed with 6 mice/group. Data were presented in scatter plots, and comparisons were implemented among multiple groups by one-way ANOVA. *P* < 0.05 indicated that the difference was statistically significant

To determine whether PFKFB4 promotes TAM M2 polarization and tumor growth via the glycolysis/H3K18la axis in vivo, Huh-7 cells were infected with lentiviruses overexpressing PFKFB4 (Lv-oe-PFKFB4) or silencing PFKFB4 (Lv-si-PFKFB4), and subsequently injected into nude mice to establish xenograft tumor models. Western blot and RT-qPCR assays found that Lv-oe-PFKFB4 increased PFKFB4 overexpression, whereas Lv-si-PFKFB4 decreased PFKFB4 expression in Huh-7 cells (Fig. 7A-B, all *P* < 0.05).

Tumor growth was monitored by measuring tumor size, tumor volume, and tumor weight. The results showed that PFKFB4 overexpression accelerated tumor growth, whereas PFKFB4 knockdown markedly suppressed tumor growth (Fig. 7C-E, all *P* < 0.05). Subsequent assessment revealed that PFKFB4 overexpression raised the level of lactate in tumor tissues, elevated the percentage of M2-polarized TAMs (F4/80^+^CD206^+^), and enhanced the protein levels of CD206, Arg-1, and H3K18la in the tumor tissues, while PFKFB4 low expression brought about the opposite outcomes (Fig. 7F-I, all *P* < 0.05). The findings further indicate that PFKFB4 in HCC facilitates TAM M2 polarization and tumor growth in vivo via the glycolysis/lactate/H3K18la axis.

## Discussion

HCC is projected to remain a substantial and challenging global public health problem in the coming decades [37]. Aberrant upregulation of PFKFB4 has been clinically associated with aggressive tumor phenotypes [38]. Herein, this study found that PFKFB4 facilitated lactate accumulation in the TME via enhanced glycolysis, and elevated M2-related gene expression patterns through H3K18la, thereby facilitating TAM M2 polarization and tumor growth in HCC.

Glycolysis is closely implicated in HCC progression, including tumor metastasis, growth, drug resistance, apoptosis resistance, and immune escape [17]. PFKFB4 stands out as a pivotal enzyme in the glycolytic pathway, with dual phosphatase and kinase activities [39]. Accumulating evidence supports the oncogenic role of PFKFB4 in various malignancies, such as gastric carcinoma and triple-negative breast carcinoma [40, 41], and elevated PFKFB4 expression has been reported in oral squamous cell, breast, and gastric cancer [39]. In this study, bioinformatics analyses identified PFKFB4 as a significantly upregulated glycolysis-related gene in HCC. Consistently, our findings unraveled increased expression of PFKFB4 in HCC and demonstrated its association with poor prognosis of HCC patients, which were partially consistent with the findings of an existing study confirming that overexpression of PFKFB4 is indicative of adverse prognosis in HCC [34]. Altogether, the findings support a tumor-promoting role of PFKFB4 in HCC and provide mechanistic insight into how aberrant PFKFB4 expression contributes to HCC progression.

Interestingly, M1 or M2 functional polarization of TAMs represents a key mechanism governing their roles in either tumor suppression or promotion [42]. During the early stages of cancer, TAMs adopt a pro-inflammatory M1 phenotype to mediate anti-tumor effects; however, the TME can drive their transition into an immunosuppressive and pro-angiogenic M2 phenotypes, thereby promoting tumor growth and evading immune surveillance [43]. In vitro study has shown that the promotion of M1 macrophage polarization can restrain the migratory, proliferative, and invasive abilities of liver cancer cells [44], whereas M2 polarization of TAMs facilitated HCC progression [45]. Despite these findings, the relationship between PFKFB4 expression and TAM infiltration in HCC remains poorly understood. Our findings observed a decrease in the proportion of M1-TAMs, and an increase in M2-TAMs in tumor tissues from patients with high PFKFB4 expression compared with those with low PFKFB4 expression. Besides, PFKFB4 expression was negatively correlated with M1-TAM infiltrationand correlated positively with M2-TAM infiltration. To our knowledge, this study provide the first evidence that PFKFB4 might influence TAM M2polarization in HCC tissues.

Distinct from normal cells, cancer cells preferentially metabolize glucose through glycolysis, leading to increased lactate production and glucose uptake [46, 47]. The Warburg effect is a hallmark of tumor metabolism and contributes to rapid drug resistance, metastasis, and proliferation in tumor cells [48]. The enhanced glycolysis stimulates the growth and metastasis of HCC cells [49]. PFKFB4 plays a key role in promoting aerobic glycolysis in carcinomas [50, 51], particularly in HCC, where it facilitates disease progression and metastasis [35]. To elaborate on this mechanism, our results showed that PFKFB4 knockdown in HCC cells declined ATP production, lactate production, and glucose uptake levels, along with decreased cell migration and proliferation, whereas PFKFB4 overexpression restored these levels. In a similar light, inhibition of PFKFB3 reduced ATP production, glucose uptake, and lactate release in endometrial cancer [52], and the lysine-specific histone demethylase 3 A axis-mediated PFKFB4 upregulation promotes aerobic glycolysis and osteosarcoma progression [53]. Furthermore, PPFIA4 has been shown to facilitate migration and proliferation in colon carcinoma cells by fortifying tumor glycolysis [54]. To conclude, PFKFB4 mediates lactate production through aerobic glycolysis thereby driving HCC cell proliferation and migration.

Arg-1 is a critical factors of TAM-mediated immunosuppression and is highly expressed in functional and immature M2 TAMs [55]. M2 polarization of macrophage is motivated by many cytokines, including IL-10, IL-4, and IL-13, as well as various signaling pathways [56]. Herein, we further explored whether PFKFB4 indirectly regulates M2 polarization of TAMs. Co-culture with PFKFB4-silenced HCC cells significantly reduced M2 markers in TAMs, including Arg-1 and CD206 protein levels, Arg-1-positive cells, and the secretion of IL-10 and IL-4, whereas co-culture with PFKFB4-overexpressing HCC cells produced the opposite effects. These results indicate that PFKFB4 can indirectly promote TAM M2 polarization through HCC cells. Notably, tumor-derived lactate produces a promotional effect on M2 polarization of TAMs [26]. Histone lactylation is perceived as a novel lactate-regulated epigenetic modification, which can engage in the regulation of M2 polarization-associated gene expression [27]. In this study, we identified increased expression of MCT1 in TAMs co-cultured with PFKFB4-overexpressing HCC cells and intensified ability of TAMs to uptake lactate, along with elevated H3K18la and PanKla levels, raised enrichment level of H3K18la on CD206 and Arg-1 promoters, and up-regulated CD206 and Arg-1 transcription levels. These outcomes were partially reversed by the inhibition of MCT1 expression in M0 macrophages or by reducing lactate in the co-culture supernatant. However, there are rare reports regarding the role of PFKFB4 in HCCs in the regulation of TAM M2 polarization through H3K18la. Here, we propose for the first time that PFKFB4 could modulate M2 polarization of macrophages via H3K18la in HCC cells. Evidence has shown that overexpression of PFKFB4 in HCC cells promotes lactate accumulation in TME, which in turn induces histone lactylation and M2 polarization in TAMs. As a signaling molecule, lactate is taken up by immune cells via the transporter MCT1 to directly regulate the pro-tumor phenotype of TAMs [57]. In our study, overexpression of PFKFB4 in HCC cells increases MCT1 expression in TAMs. Furthermore, the inhibition of MCT1 in macrophages abolished the PFKFB4-induced increase in histone lactylation and M2 polarization during co-culture. This may be mediated by other factors secreted by PFKFB4-overexpressing HCC cells that influence MCT1 expression in macrophages. The specific molecular regulatory mechanism underlying this PFKFB4-driven MCT1 upregulation in TAMs remains to be elucidated and warrants further investigation. Furthermore, we also conducted in vivo verifications in nude mice and found consistent results that PFKFB4 could amplify M2 polarization of TAMs via the glycolysis/H3K18la pathway, thus expediting tumor growth in HCC.

Previous studies have established that PFKFB4 promotes glycolysis and tumor progression in HCC [58, 59]. Our study reveals, for the first time, that PFKFB4-induced lactate accumulation promotes H3K18la levels in TAMs. This epigenetic modification transcriptionally activates M2 polarization‑related genes, such as Arg-1 and CD206, thereby driving TAMs toward an M2-polarized phenotype and facilitating HCC tumor growth. These findings uncover the previously uncharacterized mechanism by which PFKFB4 influences TAM M2 polarization in HCC and provide a novel link between lactate metabolism and chromatin-level epigenetic regulation in TAMs. However, we primarily focused on elucidating the molecular mechanisms by which PFKFB4 influences TAM M2 polarization in HCC. Our clinical data served as a preliminary verification of these mechanism findings, providing complementary evidence rather than forming the primary foundation of the core conclusions. This experimental framework aligns with established methodologies that integrate modest clinical cohorts with rigorous in vivo and in vitro models to validate mechanisms [60, 61]. Nevertheless, several limitations warrant acknowledgement. First, the modest clinical sample size represents a study limitation; we plan to address this through multi-center collaborations to further validate clinical relevance. Second, the absence of T cells in nude mice restricts the biological investigation of TAMs and the complex immune microenvironment crosstalk. Future studies utilizing immunocompetent animal models are essential for a more holistic understanding of these interactions. Additionally, cell-specific mechanisms, such as the subcellular localization of PFKFB4 within cancer cells, remain to be further investigated. In addition, due to other genes being also involved in the regulation of glycolysis in HCC, it is ambiguous whether PFKFB4 plays a major role. Also, there are various potential downstream target genes regulated by H3K18la in TAMs, and it is unknown whether H3K18la regulates the expression of other M2 polarization-related genes to affect M2 polarization. As a consequence, these are our future directions to address this question in depth.

## Supplementary Information

Below is the link to the electronic supplementary material.

Supplementary Material 1: Detection of mycoplasma contamination in cell samples

Supplementary Material 2: Detection of the number of CD68-positive cells by flow cytometry

Supplementary Material 3

## Data Availability

The data that support the findings of this study are available from the corresponding author upon reasonable request.
