# Supplementary figures and images for: PFKFB4 promotes M2 polarization of tumor-associated macrophages through aerobic glycolysis-mediated modification of histone H3K18 lactylation in hepatocellular carcinoma
Source: Cancer Metab. 2026 Apr 15;14:15. doi: 10.1186/s40170-026-00431-8 (PMC13182095; doi:10.1186/s40170-026-00431-8)

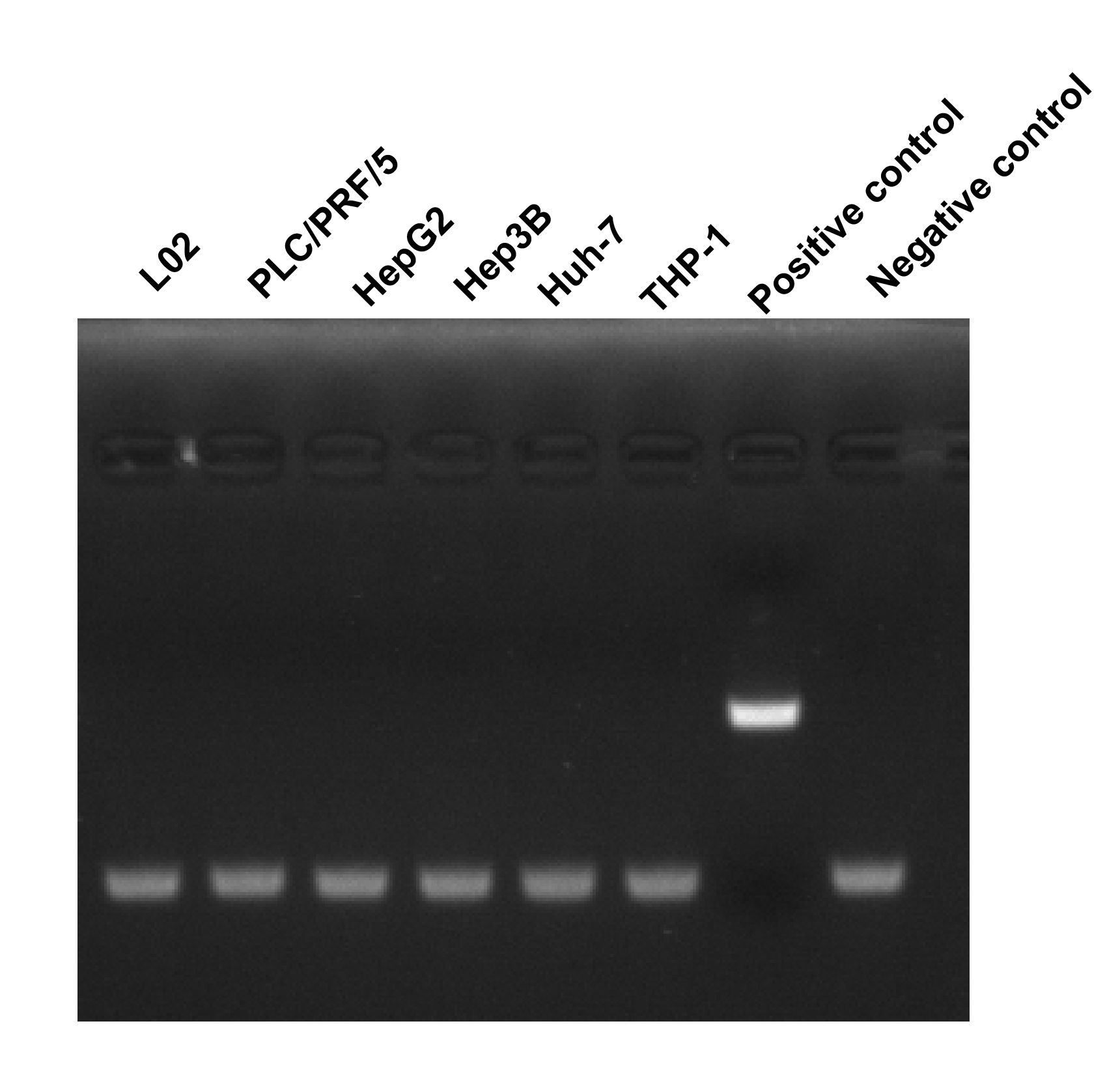

Supplement: Supplementary file 1 — Supplementary Material 1: Detection of mycoplasma contamination in cell samples [file 40170_2026_431_MOESM1_ESM.tif]

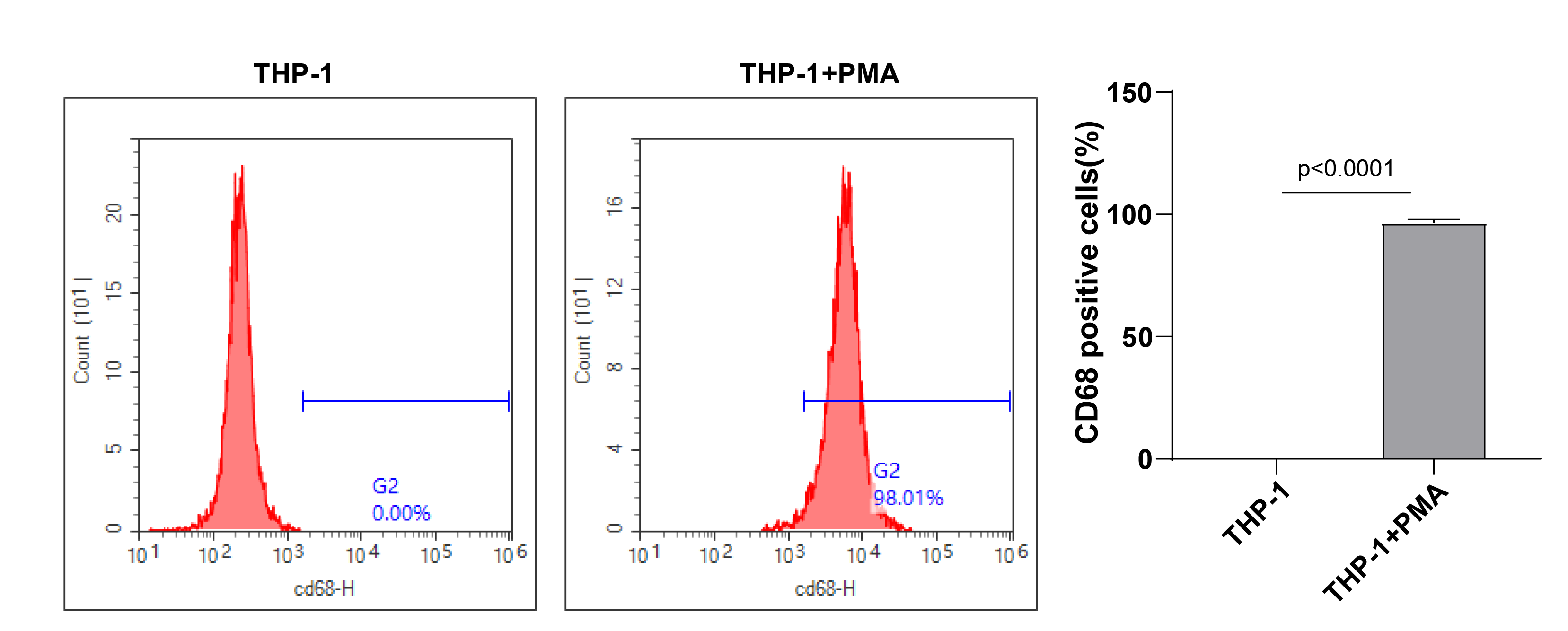

Supplement: Supplementary file 2 — Supplementary Material 2: Detection of the number of CD68-positive cells by flow cytometry [file 40170_2026_431_MOESM2_ESM.tif]
