# Supplementary material for: PFKFB4 promotes M2 polarization of tumor-associated macrophages through aerobic glycolysis-mediated modification of histone H3K18 lactylation in hepatocellular carcinoma
Source: Cancer Metab. 2026 Apr 15;14:15. doi: 10.1186/s40170-026-00431-8 (PMC13182095; doi:10.1186/s40170-026-00431-8)
Supplement: Supplementary file 3 — Supplementary Material 3 [file 40170_2026_431_MOESM3_ESM.docx]

**Supplementary table 1** Clinical baseline data of HCC patients

| Items | | HCC (n = 30) |
| --- | --- | --- |
| Age (years) | | 58.7 ± 5.4 |
| Sex [n, (%)] | | 22 (73.33) |
| Smoking history [n, (%)] | | 5 (16.67) |
| Drinking history [n, (%)] | | 4 (13.33) |
| BMI | | 25.3 ± 3.6 |
| Creatinine (mg/dL) | | 1.0 ± 0.2 |
| ALT (U/L) | | 79.0 ± 31.0 |
| AST (U/L) | | 83.6 ± 29.1 |
| ALP (U/L) | | 135.1 ± 39.2 |
| GGT (U/L) | | 99.1 ± 43.7 |
| Albumin (g/dL) | | 4.6 ± 0.7 |
| AFP (ng/mL) | ≤ 400 | 10 (33.33) |
|  | > 400 | 20 (66.67) |
| Tumor size (cm) | ≤ 5 | 17 (56.67) |
|  | > 5 | 13 (43.33) |
| TNM stage [n, (%)] | I | 12 (40.00) |
|  | II | 8 (26.67) |
|  | III | 10 (33.33) |
|  | IV | 0 (0.00) |
| Recurrence [n, (%)] | | 16 (53.33) |

Note: BMI, body mass index; AST, aspartate transaminase; ALT, alanine transaminase; ALP, alkaline phosphatase; GGT, gamma-glutamyl transferase; AFP, alpha-fetoprotein. Enumeration data were presented as cases and percentages and were compared using the chi-square test. Measurement data conforming to a normal distribution were expressed as mean ± standard deviation. Comparisons between groups were conducted using the independent samples *t*-test. A *P* value < 0.05 was considered statistically significant.
